# Hypothalamic FTO is associated with the regulation of energy intake not feeding reward

**DOI:** 10.1186/1471-2202-10-129

**Published:** 2009-10-27

**Authors:** Pawel K Olszewski, Robert Fredriksson, Agnieszka M Olszewska, Olga Stephansson, Johan Alsiö, Katarzyna J Radomska, Allen S Levine, Helgi B Schiöth

**Affiliations:** 1 Department of Neuroscience, Functional Pharmacology, Uppsala University, BMC, 75124 Uppsala, Sweden; 2 Minnesota Obesity Center, Saint Paul, MN 55108, USA; 3 Department of Food Science and Nutrition, Saint Paul, MN 55108, USA

## Abstract

**Background:**

Polymorphism in the FTO gene is strongly associated with obesity, but little is known about the molecular bases of this relationship. We investigated whether hypothalamic FTO is involved in energy-dependent overconsumption of food. We determined FTO mRNA levels in rodent models of short- and long-term intake of palatable fat or sugar, deprivation, diet-induced increase in body weight, baseline preference for fat versus sugar as well as in same-weight animals differing in the inherent propensity to eat calories especially upon availability of diverse diets, using quantitative PCR. FTO gene expression was also studied in organotypic hypothalamic cultures treated with anorexigenic amino acid, leucine. In situ hybridization (ISH) was utilized to study FTO signal in reward- and hunger-related sites, colocalization with anorexigenic oxytocin, and c-Fos immunoreactivity in FTO cells at initiation and termination of a meal.

**Results:**

Deprivation upregulated FTO mRNA, while leucine downregulated it. Consumption of palatable diets or macronutrient preference did not affect FTO expression. However, the propensity to ingest more energy without an effect on body weight was associated with lower FTO mRNA levels. We found that 4-fold higher number of FTO cells displayed c-Fos at meal termination as compared to initiation in the paraventricular and arcuate nuclei of re-fed mice. Moreover, ISH showed that FTO is present mainly in hunger-related sites and it shows a high degree of colocalization with anorexigenic oxytocin.

**Conclusion:**

We conclude that FTO mRNA is present mainly in sites related to hunger/satiation control; changes in hypothalamic FTO expression are associated with cues related to energy intake rather than feeding reward. In line with that, neurons involved in feeding termination express FTO. Interestingly, baseline FTO expression appears linked not only with energy intake but also energy metabolism.

## Background

Human studies show a strong association of a single nucleotide polymorphism in the FTO gene with obesity and diabetes in diverse ethnic groups [1-7]. Common intronic variants in this gene have been linked with increased body weight and adiposity [8]; e.g., individuals homozygous for the rs9939609 allele, weigh ~3 kg more than those with low-risk alleles [3]. High-risk variants carry a population-attributable likelihood of overweight and obesity exceeding 20% [3,6]. Aside from the association with BMI, variations in the FTO gene have been linked with glucose metabolism [1,9], type 2 diabetes [10,11], distribution of subcutaneous fat and amount of liver fat, as well as non-visceral and visceral adipose tissue [12]. FTO codes for 2-oxoglutarate-dependent nucleic acid demethylase that likely plays a role in oxidative demethylation of 3-methylthymine and 3-methyluracil in single-stranded DNA and RNA [13].

Little is known about the nature of the association between FTO and BMI; only a few basic research studies have been performed on laboratory animals. Fontanesi et al. found that FTO is associated with intermuscular fat deposition in pigs [14]. FTO mRNA is present in many tissue types, but its most abundant expression was detected in the brain, particularly, in hypothalamic nuclei which govern feeding [15,16]. In rodents, starvation modifies FTO mRNA levels detected with real-time PCR (rtPCR) in the entire hypothalamus and in the arcuate nucleus (ARC), however both up- and downregulation have been shown, thus, FTO expression alterations may be dependent on the severity of starvation or other factors [16,17,15]. These changes are independent from leptin [16]. Generation of the knock-out (KO) model was a crucial step in understanding FTO's role in body weight control. KO mice displayed decreased body weight despite consuming more standard chow than wild type (WT) controls; this effect persisted from post-natal day 2 onwards. In addition, FTO mRNA was found to colocalize with ARC proopiomelanocortin (POMC) [18], which gives rise to, among others, a satiety factor alpha-melanocyte stimulating hormone and reward mediating beta-endorphin.

Animal experiments performed to date have not addressed the key issue of whether FTO's involvement in weight gain is based solely on energy-related mechanisms or there is also a reward component. The current project was designed to characterize the relationship between FTO and obesity through feeding models, molecular analyses and functional neuroanatomy in mice. Special attention was given to the hypothalamus as - aside from primarily regulating energy intake - it mediates feeding reward [19,20].

We investigated whether FTO mRNA levels respond to a brief fast. Since overeating, especially of palatable tastants, serves as an obesogenic factor, we studied the effect of short-term (48 h) overconsumption of sucrose or fat on hypothalamic FTO expression. We examined whether body weight increase induced by long-term exposure to sugar has any effect. Furthermore, individual preferences for fat versus sucrose were correlated to the baseline FTO expression. Since same-weight mice exhibited inherent differences in their propensity to consume energy especially when diverse diets were offered, we compared the baseline expression profiles in individuals that ate more to the ones that ingested fewer calories and we employed a similar rat model to confirm our findings. Organotypic cultures of the hypothalamus were used to study FTO mRNA levels following the addition of a satiating amino acid, leucine [21].

We performed in situ hybridization (ISH) to compare FTO expression in strictly reward-related sites to that in the hypothalamus, a region involved in both feeding for energy and reward [19,20]. As FTO was found to coexpress with POMC, double ISH-immunohistochemistry (IHC) was performed to determine whether FTO colocalizes with another hypothalamic feeding regulator, the anorexigenic peptide, oxytocin [22,23]. Finally, using IHC detection of an immediate-early gene product, c-Fos, we studied activity of FTO neurons at initiation vs. termination of feeding.

## Methods

### Animals

Male C57BL/6J mice (Scanbur, Sweden) were housed individually or in groups of two (PCR Exp. 1, 2 and 3) in macrolon cages with LD 12:12 (lights on at 0700). The animals were 12 weeks old and they weighed ca. 28 g at the beginning of the experiment. Water and chow (Lactamin, Sweden) were available ad libitum unless noted otherwise. Procedures described herein received a prior approval from the Uppsala Animal Welfare Committee.

### Rt-PCR studies

#### Experiment 1. Hypothalamic FTO expression following 16-h deprivation

Food was removed just before the onset of darkness and mice (n = 8) were decapitated between 1000 and 1100 on the next day. The controls (n = 8) had food ad libitum.

#### Experiment 2. Hypothalamic FTO expression following 48-h consumption of palatable sucrose or Intralipid

Mice were given a bottle of 10% sucrose or 4.1% Intralipid in addition to chow for 48 h; controls had chow only (n = 8/group). Intralipid (Fresenius, Sweden), a palatable lipid emulsion of soybean oil, glycerol and egg yolk phospholipids, has been used in experiments utilizing liquid diets [24,25]. Sucrose and Intralipid were isocaloric (0.4 kcal/g), whereas the energy content of chow was 3.6 kcal/g. The solutions were similar in palatability: each mouse ingested on average 8.1 kcal of Intralipid and 7.4 kcal of sucrose daily. Total caloric intake per mouse was 10.3 kcal in the chow group, 14.1 kcal in the sucrose group and 12.9 kcal in the Intralipid group. Mice were decapitated after 48 h (between 1100 and 1200).

#### Experiment 3. Hypothalamic FTO expression upon increased body weight

Mice were given 10% sucrose in addition to chow for 3 weeks; controls had chow only (n = 8/group). The initial body weights did not differ (controls: 27.6 ± 0.3 g; sucrose: 27.8 ± 0.5 g). After 3 weeks of chow and sucrose intake, mice weighed 32.1 ± 0.4 g, whereas the chow controls weighed 29.5 ± 0.6 g (P < 0.05; t-test). Energy intake was similar to that described for the corresponding groups in Exp. 1. Mice were decapitated after 3 weeks (1100-1200).

#### Experiment 4. Hypothalamic FTO expression in mice differing in preference for sugar vs. fat

Mice were given 10% sucrose and 4.1% Intralipid in addition to chow for 7 days. During the first two days, animals got accustomed to tastants. Subsequently, according to the standards applied in previous reports [26,27], the mice were divided based on the 5-day preference profile: 1) sucrose-preferring (n = 7), 57.0 ± 2.2% of sucrose + Intralipid calories came from sucrose; (2) fat-preferring (n = 7), 39.2 ± 3.0% calories came from sucrose; and (3) neutral (n = 7), sucrose was the source of 46.9 ± 1.9% calories. Sucrose and Intralipid were later removed and only chow was given for 21 days ("washout") so that gene expression levels at endpoint were not affected by consumption of palatable tastants. The animals were decapitated (1100-1200). Body weights at the end of the palatable tastant availability and at the end of the "washout" phase did not differ between the preference groups.

#### Experiment 5. Hypothalamic FTO expression in mice differing in calorie intake upon availability of a diverse diet

The set-up in Exp. 4 provided a diverse diet that included standard chow, palatable sucrose and Intralipid. Although animals displayed different propensities to ingest sucrose and Intralipid, some of them - regardless of preference - ingested overall (sucrose+Intralipid+chow) more energy than others. The "small eaters" (n = 10) ate daily 11.3 ± 0.4 kcal of all tastants while the "big eaters" (n = 11), 14.4 ± 1.2 kcal (P < 0.05; t-test). The difference in the amount of consumed food was significant in the environment rich in diverse foods. The initial body weight did not differ between the groups ("small eaters": 27.2 ± 0.4 g; "big eaters": 27.5 ± 0.3 g). The 7-day exposure to palatable diets did not induce changes in body weight (29.0 ± 0.4 g and 28.3 ± 0.3 g, respectively; P = 0.287). Once palatable liquids were removed and the animals had only access to chow for 21 days, energy intake did not differ between groups during this "washout phase" although a slight trend remained (cumulative intake for big eaters: 241.8 ± 14.1 kcal vs small eaters: 221 ± 12.6 kcal; P = 0.160) and neither did body weights (P = 0.605) at decapitation.

#### Experiment 6. Arcuate hypothalamic FTO expression in rats that differ in calorie intake upon availability of a diverse diet

This experiment was included to examine whether the FTO expression pattern in the "big/small eaters" paradigm can be detected in rats. Male Wistar rats, housed individually with free access to chow and water, received for 10 days a 30% sucrose solution (n = 10), a bowl of lard (n = 10), or both tastants (n = 15). Animals increased their caloric intake when presented with the palatable diets (229 ± 3.4 kcal/kg on chow and 276 ± 5.7 kcal/kg on palatable diets; P < 0.0001). The animals were divided into "big eaters" (n = 17) and "small eaters" (n = 17): 249 ± 3.0 kcal/kg for the "small eaters" and 302 ± 5.9 kcal/kg for the "big eaters". Body weights did not differ between the groups following the exposure to palatable diets or at endpoint (P = 0.411 and 0.640, respectively). Arcuate hypothalamic FTO expression was studied with rtPCR.

#### Experiment 7: FTO expression in organotypic cultures of the hypothalamus treated with anorexigenic leucine

Organotypic hypothalamic slice cultures were prepared as previously described [28]. Briefly, 21-day-old male C57BL6 mice were decapitated, the brains were removed under sterile conditions and transferred to the Gey's Balanced Salt Solution (GBSS) (Biological Industries, Israel) containing 25 mM glucose. Hypothalami were dissected and sliced into 300-μm thick sections using the McIlwian tissue chopper. Slices from each brain were placed separately on semiporous membranes (Millipore) inserted into six-well culture plates (Sarstedt) containing 1.2 ml of the serum-Opti-MEM medium in each well. Culture medium was a mixture of Opti-MEM (50%; Gibco), horse serum (25%) and Hanks' Balanced Salt Solution (HBSS) (25%; Gibco) supplemented with 50% D(+) glucose monohydrate (25 mM, Merc). Growing medium of the experimental group (n = 7) was supplemented with 0.3 μg leucine; controls (n = 7) had no leucine added. The amount of supplemented leucine was based on the previous report utilizing central administration of this amino acid to reduce food intake [21]. Cultures were maintained at 37°C in a humidified atmosphere of 5% CO_2_. Medium was changed daily. After 48 h FTO expression was studied with rtPCR.

### PCR

Brains were excised, hypothalami dissected, immersed in RNAlater (Ambion, USA), kept at room temperature for 2 h and, thereafter, stored at -80°C until further processed. Samples were homogenized and RNA prepared as previously described [15,29] [see Additional file 1].

### RT-PCR

Relative expression levels of 6 housekeeping genes (HKGs) and genes of interest were determined with quantitative rtPCR. Each reaction, with a total volume of 20 μl, contained 20 mM Tris/HCl, 50 mM KCl, 4 mM MgCl_2_, 0.2 mM dNTP, DMSO (1:20) and SYBR Green (1:50000). Template concentration was 5 ng/μl and the concentration of each primer was 2 pmol/μl. Primers were designed with Beacon Designer (Premier Biosoft) using the SYBR Green settings. Sequences are presented in Additional file 1. All rtPCR experiments were performed in duplicates; a negative control for each primer pair and a positive control with 5 ng/μl of genomic DNA were included on each plate. Amplifications were performed with 0.02 μ/ml Taq DNA polymerase (Biotools, Sweden) under the following conditions: initial denaturation at 95°C for 3 min, 50 cycles of denaturing at 95°C for 15 sec, annealing at 52.8-60.1°C for 15 sec and extension at 72°C for 30 sec.

### Data analysis and relative expression calculations

Analysis of rtPCR data was performed as previously reported [29]. MyIQ 1.0 software (Bio-Rad) was used. Primer efficiencies were calculated using LinRegPCR [30] and samples were corrected for differences in primer efficiencies. The GeNorm protocol described by Vandesompele et al. [31] was used to calculate normalization factors from the expression levels of the HKGs. Grubbs' test was used to remove outliers (6 outliers were removed) [32,33]. Differences in gene expression between groups were analyzed with ANOVA followed by Fisher's PLSD test where appropriate. P < 0.05 was used as the criterion of statistical significance.

### ISH detection of FTO in sites involved in hunger versus reward

Four mice were anaesthetized with pentobarbital and perfused with 5 ml 0.9% NaCl followed by 50 ml 4% paraformaldehyde in 0.1 M phosphate buffer. Perfusions took place between 1100 and 1200. The brains were immersed in the same fixative overnight at 4°C. Coronal sections were cut at 50 μm with a Vibratome and processed as free-floating sections.

The FTO cDNA clone was obtained from Invitrogen. Plasmid DNA preparation was done with the JETstar 2.0 Plasmid Purification Midi Kit/50 (Genomed, Germany). The clones were sequenced (MWG, Germany) and confirmed to contain 773 bp of the 3' UTR of the mouse FTO transcript. 20 μg of plasmid DNA was lineraized by digestion with 30 U SpeI (Fermentas, Latvia) for 3 h. Ribo probes were synthesized using 1 μg template, RiboLock Ribonuclease Inhibitor (Fermentas, Sweden) and 40 U RNA polymerases in the presence of digoxigenin-11-UTP (Roche). Probe quantification was performed with a NanoDrop ND-1000 Spectrophotometer (NanoDrop Technologies). The synthesis of the FTO RNA probe was performed according to the previously described protocol [15]. FTO mRNA was detected in the tissue as described before [15] and the full protocol can be found in Additional file 1. Evaluation of FTO expression was based on the visual inspection; the following scale was applied: 0, not detectable; +, weak; ++, moderate; +++, high. Every other section was used for the ISH.

### Detection of oxytocin in FTO neurons

Following the completion of the ISH procedure, every other PVN section was used in the IHC staining to detect oxytocin. Sections were rinsed in TBS, incubated in 3% H_2_O_2_/10% methanol for 10 min. The tissue was incubated 24 h in the rabbit-anti-oxytocin antibody (1:15000; Chemicon), followed by incubations in the goat-anti-rabbit (1:400; Vector Laboratories) and avidin-biotin complex (1:800; Vector Laboratories) for 1 h. Peroxidase in the tissue was visualized with 0.05% diaminobenzidine and 0.01% H_2_O_2 _was used to catalyze the reaction. Rinsing steps were done with TBS. The vehicle for antibody incubations was 0.25% gelatin and 0.5% Triton X-100 in TBS. Sections were analyzed visually and the percentage of oxytocin neurons colocalizing with FTO was determined.

### Detection of c-Fos in FTO neurons at feeding initiation and termination

Mice (n = 10) were food-deprived for 16 h. Chow was returned at 1000. Deprivation-induced meal was completed within ~30 min (each mouse ingested 2.0-2.4 g chow). Five mice were perfused at the time corresponding to the initiation of feeding, whereas the other five at the time corresponding to the termination of consumption. Since the maximum c-Fos IR occurs 1 h after the onset of neuronal activity [34], animals were perfused 1 h after food presentation (initiation)or 1 h after meal completion (termination).

The ISH protocol was as described above. In IHC, the modifications included the use of the goat-anti-Fos (1:1500; Santa Cruz) and rabbit-anti-goat (1:400; Vector Laboratories) antibody. The percentage of Fos-positive FTO in the PVN and ARC was determined and values were compared with Student's t-test (significant when P < 0.05).

## Results

**Figure 1:**
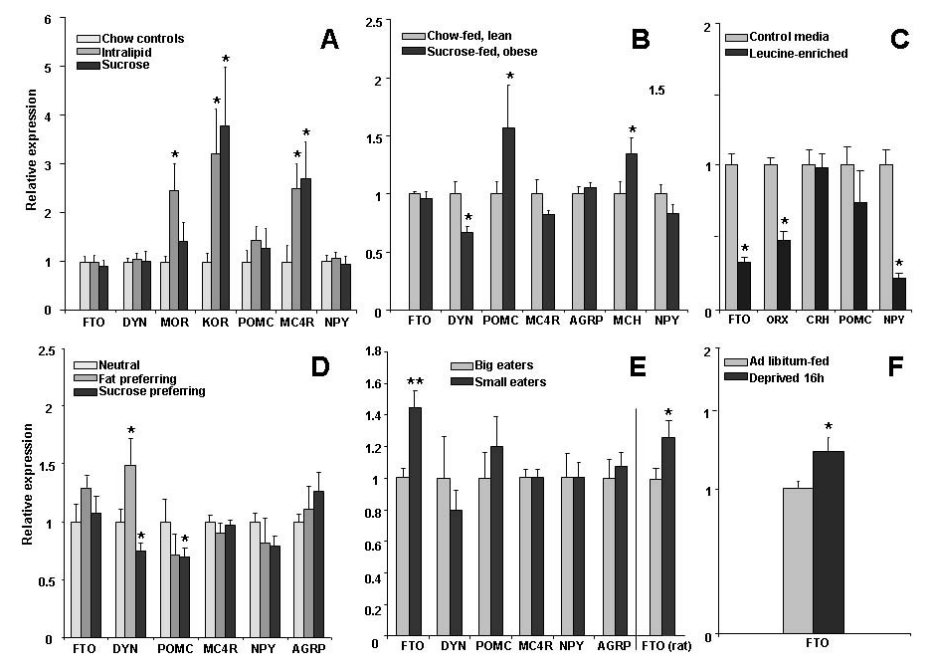
**Relative expression of FTO and feeding-related genes in the hypothalamus of mice**. **A**. Mice were exposed for 48 h to palatable Intralipid or sucrose solutions in addition to chow (n = 8/group). **B**. Mice differed in body weight by ca. 2.6 g. Increased body weight was induced by 3-week exposure to the sucrose solution given in addition to chow (n = 8/group). **C**. Organotypic cultures of the hypothalamus were treated for 48 h with anorexigenic leucine versus untreated controls (n = 7/group). **D**. Mice differed in 5-day preference for fat (Intralipid) vs. sucrose (n = 7 per preference-based group). **E**. Mice differed in their propensity to ingest more calories during exposure to palatable diets ("big eaters", vs. "small eaters"; n = 11 and 10, respectively). A similar rat model was used as control of FTO expression in another species (FTO rat). **F**. Mice were deprived of food for 16 h preceding decapitation (n = 8/group). AGRP, Agouti-related protein; DYN, dynorphin; MC4R, melanocortin receptor-4; NPY, neuropeptide Y; ORX, orexin; POMC, proopiomelanocortin; KOR, kappa opioid receptor; MOR mu receptor; MCH, melanin concentrating hormone. * - P < 0.05; ** - P < 0.01.

Exposure to palatable, isocaloric sucrose or Intralipid for 48 h did not affect FTO expression (Fig. 1a); the animals had the same body weight. When mice were given 3-week access to sugar, which caused a greater body weight increase than intake of chow alone (32.1 ± 0.4 g and 29.5 ± 0.6 g, respectively; P < 0.05), FTO mRNA levels were still the same in both groups (Fig. 1b). Also animals that had distinct preference profiles for sugar vs. fat did not differ in the baseline FTO expression (Fig. 1d). In the three aforementioned models, changes in expression of other reference genes known for their involvement in food intake control could be detected. Those included kappa (KOR) and mu (MOR) opioid receptors, dynorphin (DYN), proopiomelanocortin (POMC), melanocortin receptor-4 (MC4R) and melanin concentrating hormone (MCH).

In contrast, FTO expression was increased in deprived animals (Fig. 1f). Treating organotypic cultures of the hypothalamus with anorexigenic leucine caused downregulation of FTO mRNA as well as of two orexigenic genes, NPY and ORX (Fig. 1c). Finally, the propensity to eat less during the exposure to a self-composed, diverse and palatable diet was associated with higher FTO mRNA levels. During the exposure, "small eaters" ate fewer calories than "big eaters" did (11.3 ± 0.4 kcal and 14.4 ± 1.2 kcal per day, respectively; P < 0.05) and, after the 3-week "washout" phase of chow intake (trend of elevated calorie intake in big eaters; P = 0.160), they had ~50% higher FTO mRNA content (P = 0.004; Fig. 1e). There was no change in expression of several reference genes, including DYN, POMC, neuropeptide Y (NPY), Agouti-related protein (AGRP) and MC4R (Fig. 1e). Applying a similar paradigm in rats resulted in the same outcome for the FTO mRNA in the hypothalamic ARC, i.e. "small eaters" had an elevated level of FTO (P = 0.030; Fig. 1e).

**Figure 2:**
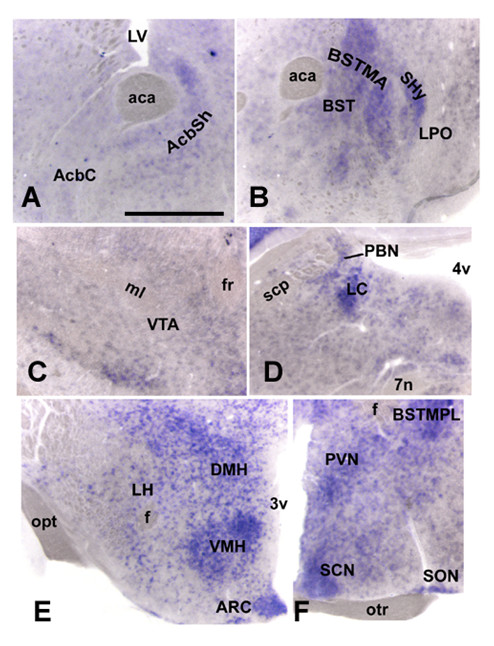
**ISH detection of FTO mRNA in sites involved in the regulation of energy- or reward-related feeding**. Panels A-D show sites affecting mainly reward, whereas panels E and F depict energy-related areas. aca, anterior commisure; AcbC, nucleus accumbens core; AcbSh, Acb shell; ARC, arcuate nucleus; BST, bed nucleus of the stria terminalis; BSTMA, BST medial anterior; BSTMPL, BST medial posterolateral; DMH, dorsomedial nucleus; f, fornix; fr, fasciculus retroflexus; LC, locus corelueus; LH, lateral hypothalamus; LPO, lateral preoptic area; LV, lateral ventricle; ml, medial lemniscus; otr, optic tract; PBN, parabrachial nucleus; PVN, paraventricular nucleus; SHy, septohypothalamic nucleus; SCN, suprachiasmatic nucleus; scp, superior cerebellar peduncle; SON, supraoptic nucleus; VMH, ventromedial nucleus; VTA, ventral tegmental area; 3v, 3rd ventricle; 4v, 4th ventricle; 7n, facial nerve. Scale bar: 0.4 mm (A, B) 0.5 mm (C-F).

**Figure 3:**
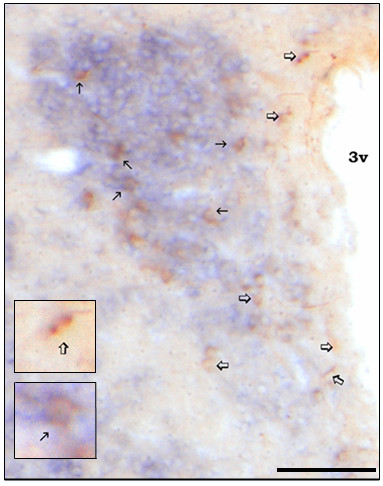
**Colocalization of FTO and oxytocin**. Oxytocin (brown cells) and FTO mRNA (blue cells) detected in the coronal section containing the PVN. Thin arrows, neurons expressing oxytocin and FTO; Open arrows, oxytocin cells devoid of FTO. Scale bar: 0.25 mm. Inserts: three-fold higher magnification of PVN neurons of interest.

**Figure 4:**
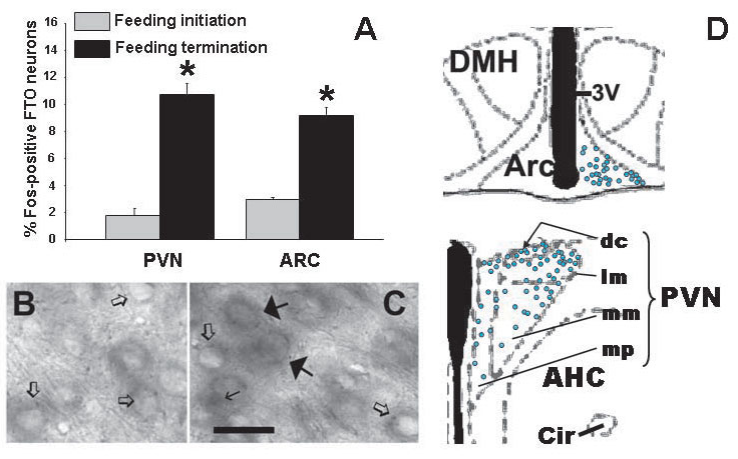
**Fos-IR FTO neurons in the PVN and ARC at initiation and termination of feeding**. c-Fos was detected by IHC, whereas FTO mRNA by ISH. **A**. Percentage of Fos-positive FTO cells in the PVN and ARC. **B **and **C**. Coronal sections of mice perfused at the beginning and at the end of a meal. Thin arrows, Fos-positive nuclei, thick solid arrows, Fos-positive FTO cells, open arrows, FTO neurons devoid of Fos. Scale bar: 0.02 mm. **D**. Schematic representation of distribution of Fos-positive FTO cells (each blue point indicates one such cell) in the representative sections encompassing the PVN and ARC. dc, dorsal cap; lm, lateral magnocellular; mm, medial magnocellular; mp, medial parvocellular.

ISH detection of FTO mRNA revealed expression primarily in the hypothalamus. Some sites linked with reward, such as the ventral tegmental area (VTA) and nucleus accumbens (Acb), had very little or lacked the FTO signal, whereas others, e.g., the bed nucleus of the stria terminalis and parabrachial nucleus, expressed moderate levels of FTO (Table 1; Fig. 2). Double ISH-IHC analysis showed that FTO mRNA is present in many neurons synthesizing the anorexigenic peptide, oxytocin: 55 ± 6.1% oxytocin neurons in the magnocellular subdivision of the PVN and 32 ± 5.8% in the parvocellular portion colocalized with FTO (Fig. 3). Interestingly, we found that approximately four times more FTO expressing cells in the PVN and ARC display c-Fos (they are "active") at the time corresponding to termination than to initiation of consummatory activity (Fig. 4). There was no difference between the two groups in the number of FTO cells (data not shown).

**Table 1:** Graded denotation of FTO expression in sites involved in reward-related consumption (regular font) vs. in those that regulate primarily energy intake (bold).

***Area***	***Expression***
Nucleus accumbens shell	+

Nucleus accumbens core	0

Ventral tegmental area	0

Parabrachial nucleus	+

Bed nucleus of the stria terminalis	+++

**Supraoptic nucleus**	+++

**Paraventricular nucleus**	++

**Dorsomedial nucleus**	++

**Ventromedial nucleus**	+++

**Arcuate nucleus**	+++

**Lateral hypothalamus**	+

*Other sites: septohypothalamic nucleus, ++, lateral preoptic area, 0; suprachiasmatic nucleus, +++; locus coeruleus, ++*.

Expression based on the visual inspection: 0, not detectable; +, weak; ++, moderate; +++, high.

## Discussion

Consuming excessive amounts of foods, which serves as the main factor in the development of obesity (and may result in diabetes), is driven by either a perceived need to acquire calories (eating for energy in response to the hungry state) or hedonic aspects of eating (eating for pleasure) or the combination of both. Our data strongly suggest that FTO, the first gene contributing to common forms of human obesity [8,35], plays a significant role in mechanisms responsible for the regulation of energy-related eating behavior.

This presumed involvement in hunger control is reflected by the neuroanatomical distribution of FTO visualized through ISH. This gene is scarcely expressed within the reward network: particularly low levels are found in the Acb and VTA which host, e.g., opioid and dopaminergic circuitry [36-39] mediating hedonics of food intake. On the other hand, very high expression was detected in hypothalamic regions, including the PVN, ARC and dorsomedial nucleus (DMH) that encompass key genes influencing hunger and satiety, such as NPY, AGRP, POMC and MC4R.

Distribution primarily outside the "reward network" does not exclude a possibility of this gene's involvement in reward processes, especially since the molecular components of the reward system (e.g. genes encoding opioid peptides and receptors) are dispersed throughout the CNS and present also in the hypothalamus [20]. In fact, intake of palatable diets, especially those containing fat or sugar, modifies expression of reward-related genes in the hypothalamus; and reward mediators, e.g., opioid ligands, injected in this structure are particularly effective in changing intake of such foods [40-42]. However, in our experiments we have not found evidence linking hypothalamic FTO with feeding reward. Ingestion of palatable sugar or fat did not affect FTO expression, which suggests that FTO mRNA levels do not change upon exposure to palatable tastants in general and there is no special relationship between FTO and a particular palatable macronutrient (fat or sugar). This was seen following short- (48 h) and long-term (3 weeks) intake of rewarding tastants. The outcome is in line with our previous findings showing the lack of correlation in expression of FTO and reward-related genes, including DYN and enkephalin, in rat deprivation models [15]. In addition, differences in animals' liking of sugar versus fat did not reflect any disparities in FTO gene activity profile. It suggests that the inherent preference for a specific macronutrient is not dependent on the baseline FTO expression in the hypothalamus. These data are of particular importance as they suggest that hypothalamic FTO's role is unrelated to eating for pleasure or inclination to choose one palatable macronutrient over another. As the mice maintained on sucrose for 3 weeks gained more weight than chow-fed controls, it also indicates that the development of overeating-driven obesity is not a causative factor in changes in FTO expression. This lack of change in FTO expression in the aforementioned models shows a striking difference from the response of other feeding-related "reference" genes studied herein, including opioids, melanocortins and MCH. Obviously, one cannot rule out the possibility that FTO outside the hypothalamus may affect or be affected by the feeding variables considered herein, including reward and preference, but hypothalamic FTO shows a strong association only with energy intake control.

Three independent studies have shown that depriving rodents of food influences FTO mRNA content in the brain [15-17]. In mice, fasting was reported to decrease expression of this gene, however, starvation applied in those studies was severe as it lasted 40-48 h [16,17]. Upregulation has been detected in rats. In the current study, a short-term, overnight fast, upregulated FTO mRNA. The data confirm that FTO is sensitive to a current energy status of the organism, probably in terms of both energy acquired and stored; but the results also point to the necessity of defining parameters accompanying deprivation of various magnitudes and timeframes that shape the FTO gene activity.

The Fos experiment showed that >4 times more FTO neurons in the PVN and ARC were "activated" at feeding termination than initiation. Activity of FTO expressing cells at the end of a meal suggests FTO's involvement in feeding inhibitory mechanisms. In fact, c-Fos IR is increased in the PVN and ARC of rodents following 1-h intake of caloric but not non-caloric tastants [43]. Furthermore, an upward trend in neuronal activity in hypothalamic cells synthesizing anorexigenic peptides has been reported: a greater percentage of Fos-positive neurons synthesizing oxytocin and alpha-melanocyte stimulating hormone can be detected at feeding termination [44,45]. Noteworthy, FTO is expressed in PVN neurons that produce oxytocin, which is released within the CNS in response to such satiation cues as increased stomach distention and high plasma osmolality. This colocalization occurs in magno- and parvocellular cells, which implies that FTO may influence the activity of oxytocin neurons that project to the posterior pituitary (magnocellular) as well as to sites linked with feeding control, including the nucleus of the solitary tract and dorsal motor nucleus of the vagus (parvocellular) [46]. Our oxytocin colocalization results along with the previous report regarding POMC [18] indicate that FTO may be involved in mechanisms that end consummatory activity, thus, those that are closely tied with meal size (therefore, amount of ingested energy) control. A previously reported correlation in the expression of FTO and energy-related gene encoding galanin-like peptide supports this concept [15].

The question remained as to whether the role of FTO in satiety-related hypothalamic neurons, such as those positive for POMC or oxytocin, is to increase or decrease their activity. Addition of leucine, which in vivo reduces feeding and activates anorexigenic neurons synthesizing POMC [21], to hypothalamic organotypic cultures produced downregulation of FTO expression. This suggests that nutrient cues mediating satiation decrease FTO mRNA levels while increasing activity of the satiety network (e.g., POMC cells), hence, there is the inverse relationship between FTO and satiety.

The first studies with the use of the FTO KO murine model showed that the role of FTO in energy intake and metabolism regulation goes beyond short-term control of feeding. Surprisingly, FTO KO mice weigh less even though they exhibit relative hyperphagia [18]. Our "big/small eaters" experiment further supports the findings obtained in the KO mice. This experiment was inspired by the observations that diversity of foods propels energy intake beyond levels required to satisfy energy needs. Interestingly, under such conditions, some individuals display a significantly higher degree of hyperphagia than others [19,45]. The "big eaters" - characterized by lower FTO expression (P = 0.004) - weighed the same as the "small eaters", even though during the several-day diverse food exposure period they ingested ca. 22% more energy and a slight trend of higher energy intake (P = 0.160) remained throughout the subsequent exposure to regular chow. Our data indicate that the higher energy intake that does not translate to increased body weight may be accompanied by downregulation of the FTO gene in the hypothalamus. This is in concert with the conclusion reached in the FTO KO mouse studies that not only feeding, but also energy metabolism and expenditure are affected by FTO.

## Conclusion

Changes in hypothalamic FTO mRNA levels are associated with a deprived state as well as with the presence of the amino acid leucine that promotes satiety and feeding termination. In addition, c-Fos IR of FTO neurons in the ARC and PVN increases at the end of consummatory activity; and FTO co-localizes with anorexigenic oxytocin in the PVN. It allows us to speculate that hypothalamic FTO may be involved in the regulation of energy intake, possibly by affecting feeding termination mechanisms. Distribution of FTO mRNA mainly in hunger/satiation-related sites and lack of changes in FTO expression upon exposure to palatable tastants call into question a possible involvement of FTO in the reward component of feeding. Finally, the inherent propensity to eat more without a change in body weight is associated with low hypothalamic FTO mRNA levels. In contrast, long-term palatability-driven overconsumption (irrespective of the aforementioned individual propensity to eat different amounts of food) that leads to increase in body weight does not affect FTO expression in the hypothalamus.

Overall, our molecular and histological data strongly link hypothalamic FTO with the regulation of feeding for calories not for reward; the molecular analyses also point to a possible role of this gene in energy metabolism.

## Authors' contributions

PKO - carried out the ISH experiments and tissue dissections, drafted the manuscript, RF - participated in the design of the molecular studies and design of the animal models, AMO - carried out double ISH-IHC staining, OS - carried out mouse PCR studies, JA - designed and carried out the rat FTO PCR studies, KJR - designed and carried out the organotypic tissue culture studies, ASL - participated in the statistical analyses and drafting the manuscript, HBS - participated in the design and coordination of the study and in drafting the manuscript. All authors read and approved the final manuscript.

## Supplementary Material

Additional file 1 **Detailed PCR and ISH methodology**. This file contains additional information pertaining to the PCR and ISH methodology applied herein. Click here for file
