# Supplementary material for: Hypothalamic FTO is associated with the regulation of energy intake not feeding reward
Source: BMC Neurosci. 2009 Oct 27;10:129. doi: 10.1186/1471-2202-10-129 (PMC2774323; doi:10.1186/1471-2202-10-129)
Supplement: Additional file 1 — Detailed PCR and ISH methodology. This file contains additional information pertaining to the PCR and ISH methodology applied herein. [file 1471-2202-10-129-S1.DOC]

**Additional file:** Detailed PCR and ISH methodology

**Samples and RNA.** Samples were homogenized by sonication in TRIzol (Invitrogen, Sweden) using a Branson sonifier (Branson Ultrasonics Corp., Germany). Chloroform was added to the homogenate, which was then centrifuged at 13000 rpm at 4 °C for 15 min. The water phase was transferred to a new tube, and RNA was precipitated with isopropanol. The pellets were washed 2 times with 75% ethanol, air dried and dissolved in 1x DNAse buffer. DNA contamination was removed by DNAse I treatment (Roche Diagnostics, Sweden) for 4 h at 37°C; DNAse I was inactivated by heating the samples at 75°C for 15 min. The absence of genomic DNA was confirmed by PCR with primers for mouse glyceraldehyde-3-phosphate dehydrogenase (GAPDH) or rat beta-tubulin (see Table 1) on the DNAse-treated RNA. RNA concentration was determined using a Nanodrop ND-1000 Spectrophotometer (NanoDrop Technologies, USA). cDNA was synthesized with MMLV reverse transcriptase (GE, Sweden), using random hexamers as primers.

**ISH protocol**. Sections were bleached for 15 min in 6% H_2_O_2_ in PBT. They were treated for 5 min in 0.5% Triton X before digestion for 15 min in 20 µg/ml proteinase K diluted in Tris-HCl. The reaction was stopped by washing with 2 mg/ml glycerol in PBT for 5 min. The tissue was postifixed in 4% formaldehyde for 25 min and washed in PBT. The sections were incubated for 2 h in the hybridization buffer at 55°C. The buffer consisted of 50% formamide, 5xSSC, 1% SDS, 10 mg/ml yeast tRNA (Sigma-Aldrich) and 10 mg/ml heparin in 0.1% DEPC. The probe (1µg/ml) was then heat-denatured in the hybridization buffer. Hybridization was performed overnight at 55°C. The sections were washed in the buffer 2 (50% formamide, 2xSSC, 0.1% Tween 20 in 0.1% DEPC) 3 x 30 min at 55°C, then washed in the buffer 3 (50% formamide, 0.2xSSC, 0.1% Tween 20 in 0.1% DEPC) 3 x 30 min at 55°C and in TBST (0.1% Tween 20 in TBS). Incubation in the blocking solution (1% blocking reagent; Roche Diagnostic, Sweden) was followed by incubation in the anti-digoxigenin-AP antibody (Roche Diagnostic) diluted 1:5000 in the blocking solution. The sections were incubated in the antibody overnight at 4°C. Sequential washes with 2 mM levamisole in TBST followed by washes with 2 mM levamisole in NTMT (100 mM NaCl, 10mM Tris-HCl pH 9.5, 50 mM MgCl2 and 0.1% Tween-20) were performed before color development of the alkaline phosphate-labeled probe with BM Purple (Roche Diagnostic). After mounting in DTG media with antifade (DABCO in glycerol and Tris) the sections were analyzed with the Olympus BX61W1 microscope.

**Table 1.** Real-time PCR primers (all supplied by Thermo Scientific). Rat primers are marked.

|  | **FORWARD** | **REVERSE** |
| --- | --- | --- |
| POMC | gaacgccatcatcaagaac | ctaagaggctagaggtcatc |
| DYN | gacaggagaggaagcaga | agcagcacacaagtcacc |
| NPY | cccttccatgtggtgatg | gacaggcagactggtggc |
| FTO | gatgtcagagcgtcagagag | aaggtcatggagtgagtgc |
| MC4R | cgctccagtaccataacatc | gaagaggacgcctgacac |
| AGRP | gcacaagagaccaggacatc | gccaacagcagaacacaac |
| MCH | ccgcaacatccttacagaag | gcacaagttatagcaacatcaag |
| KOR | caccttgctgatcccaaac | ttcccaagtcaccgtcag |
| MOR | cctgccgctcttctctgg | cggactcggtaggctgtaac |
| GAPDH | gccttccgtgttcctacc | gcctgcttcaccaccttc |
| FTO (rat) | aacaccaggctcttcacc | cacttcatcatcgcaggac |
| tub b (rat) | gctcaccacgccaacctac | gccgtaaactgctcagagatgc |
